# A Method for Conveying Confidence in iNaturalist Observations: A Case Study Using Non‐Native Marine Species

**DOI:** 10.1002/ece3.70376

**Published:** 2024-10-08

**Authors:** Sarah J. Ackland, David M. Richardson, Tamara B. Robinson

**Affiliations:** ^1^ Department of Botany and Zoology, Centre for Invasion Biology Stellenbosch University Matieland South Africa

**Keywords:** alien species, biological invasions, citizen science, data accuracy, data verification, monitoring

## Abstract

Concerns and limitations relating to data quality, reliability and accuracy hamper the use of citizen science initiatives in research and conservation. Valued for their cost‐effective and large data acquisition potential, citizen science platforms such as iNaturalist have been highlighted as beneficial tools to supplement monitoring using traditional data sources. However, intrinsic uncertainties in unverified observations stem from the nature of species being identified, the quality of uploaded media and georeferencing; these factors can limit the value of the data as they can result in inaccurate records. Verification of data prior to use is critical. This process can, however, be laborious and time‐consuming, with bias associated with the individual responsible for the task. To address this challenge this study developed a protocol for assigning confidence in iNaturalist observations, using marine alien and cryptogenic species observations from South Africa as a case study. A positive relationship was found between the accuracy of observations and confidence score. The inherent data quality assessment in iNaturalist, termed quality grade, was found to be an inadequate proxy for accuracy. The results of this study highlight the importance of the expert verification phase when using citizen science data. The confidence score facilitates a streamlined approach to the verification process by reducing the time taken to validate records, while assessing the three levels of uncertainty within observations and reducing researcher bias. It is recommended that this confidence score be used as an essential tool when using citizen science derived data.

## Graphical abstract

Data quality, reliability and accuracy have long been recognised as barriers to unlocking the full potential of citizen science derived data. This study developed a score to assign confidence in iNaturalist observations and streamline the verification process.

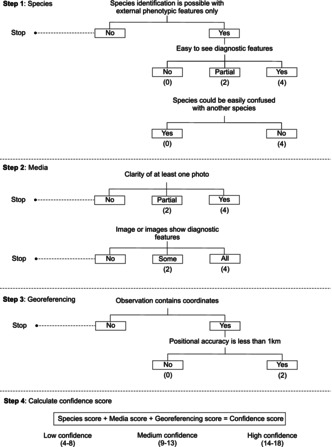

## 1 Introduction

The terms ‘citizen science’ and ‘community science’ refer to approaches that incorporate contributions from non‐scientists into scientific investigations (Munzi, Isocrono, and Ravera 2023). Growing at an exponential rate, citizen science initiatives contribute huge amounts of data that potentially contribute to an improved understanding of a variety of complex research questions across many fields (Callaghan et al. 2022; Geurts, Reynolds, and Starzomski 2023). Public engagement in the acquisition of data, especially within the field of ecology, has increased in popularity over the last decade (Pocock et al. 2017; Munzi, Isocrono, and Ravera 2023). Open‐access platforms that collect information on species occurrences have revolutionised the manner in which biodiversity data is collected (Hochmair et al. 2020). Moreover, technological advancements have enabled the amplification of data collection across broad temporal and geographical scales, maximising biodiversity and ecological data acquisition (Pocock et al. 2018; Zhang 2020; Geurts, Reynolds, and Starzomski 2023).

Accelerating global change is negatively impacting ecosystems across the planet (Corelli et al. 2024). Driven by anthropogenic drivers such as biological invasions and climate change, the cascading effects of global change threaten ecosystems and the services that they provide (Johnson et al. 2024). Evidence‐based actions that require long‐term datasets and foundational information on species distributions are required to tackle challenges associated with global change (Gonzalez, Chase, and O'Connor 2023; Johnson et al. 2024). However, traditional monitoring is costly and time‐consuming (Ahmed et al. 2022). This highlights the value of crowdsourced data (Delaney et al. 2008). iNaturalist is one of the most popular global citizen science platforms (Hochmair et al. 2020; Aristeidou et al. 2021; Barbato et al. 2021). Aimed at documenting species occurrences, iNaturalist is a social network founded on the concept of mapping and sharing biodiversity observations within a global community (Munzi, Isocrono, and Ravera 2023). Participants on the platform, termed ‘observers’, submit time‐stamped media (e.g., images or videos) of organisms along with associated metadata including geographic information, taxonomic identification and any associated descriptions (Munzi, Isocrono, and Ravera 2023). The iNaturalist community then confirms or challenges taxonomic identifications. Along with community verification, iNaturalist uses integrated artificial intelligence to provide automated taxon identification suggestions (Munzi, Isocrono, and Ravera 2023). Observations on the platform are subject to data quality assessments that determine their reliability by interrogating data completeness, suitability and accuracy (Barbato et al. 2021). This process culminates in observations being filtered according to their ‘quality grade’ before being integrated into biodiversity databases (e.g., Global Biodiversity Information Facility [GBIF] [https://www.gbif.org/]).

The quality, reliability and accuracy of data are long‐standing concerns associated with data derived from citizen science initiatives (e.g., Kosmala et al. 2016; Wittmann, Girman, and Crocker 2019). Data from these platforms are often considered as low quality and unreliable compared to data from traditional sources such as herbarium and museum collections (Burgess et al. 2017). However, some studies have found the quality of data collected by citizen scientists to be comparable to that collected by experts (Lewandowski and Specht 2015; Falk et al. 2019). The quality of data obtained from citizen science initiatives depends on many factors including participant experience and skill, task difficulty (Kosmala et al. 2016), and the uncontrollable and unpredictable nature of the citizen science data collection process. Thus, if the full value of community‐derived data is to be unlocked by researchers and conservation practitioners, limitations due to accuracy and uncertainty associated with such data must be minimised (Falk et al. 2019; Barbato et al. 2021).

iNaturalist observations have three inherent sources of uncertainty that can cast doubt on the accuracy of an observation. Firstly, some species are intrinsically challenging to identify. This can be due to species‐specific diagnostic traits that can be difficult to recognise, as well as whether or not the species could be easily confused with another species (Barbato et al. 2021). Other challenges with identification stem from a species being small; having diagnostic characters that require dissection to be visible; or lacking defining morphometric characters (in which case species‐level identification may only be achievable via molecular analysis) (Barbato et al. 2021; McMullin and Allen 2022; Munzi, Isocrono, and Ravera 2023). Secondly, uncertainty can stem from the quality of the uploaded media. Image quality is a problem in many citizen science initiatives (Falk et al. 2019), as poor‐quality images or those that do not show key diagnostic features hinder identification to species level (Barbato et al. 2021). Finally, many citizen science initiatives utilise biodiversity data for monitoring, making the spatial accuracy of observations critical. If imprecision and errors arise with georeferencing, overestimations of ecological niches and false positives of range and distribution shifts may occur (Contreras‐Díaz et al. 2023).

Verification by taxonomic experts has been highlighted as an essential part of all research that incorporates iNaturalist data (Falk et al. 2019; Barbato et al. 2021; Munzi, Isocrono, and Ravera 2023). This process can be tedious and time consuming, with accuracy being dependant on the individual validating the observations and the taxon under consideration. The aim of this study was to develop a score that can be used by researchers to assign confidence in iNaturalist observations, using marine alien and cryptogenic species observations from South Africa as a case study.

## 2 Methods

**FIGURE 1:**
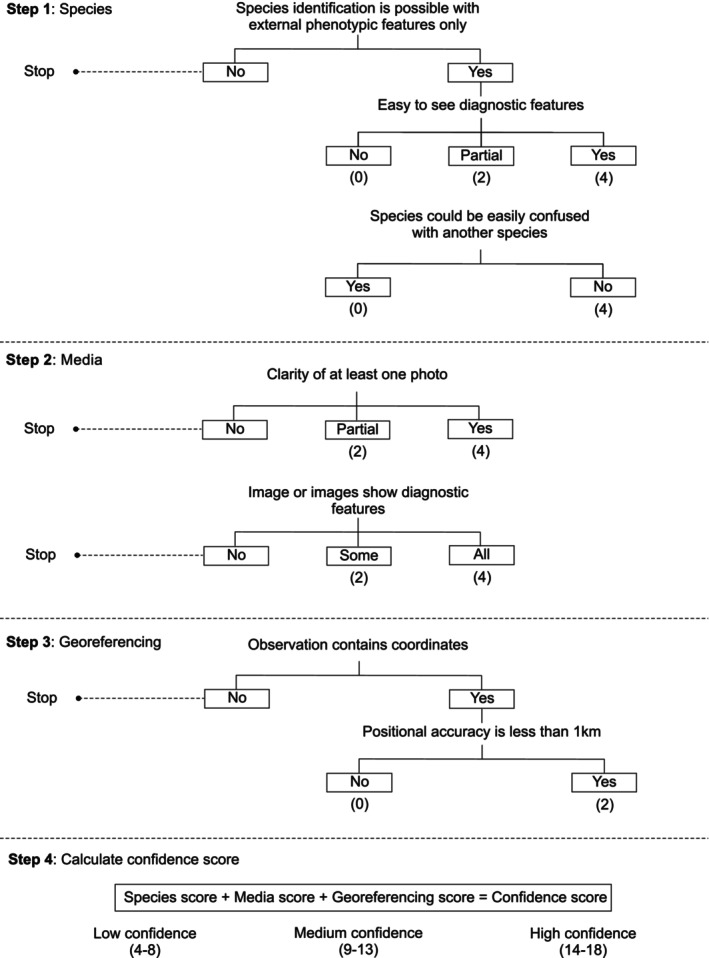
The four step process for scoring confidence of iNaturalist observations. Scores are summed to produce a confidence score reflecting low, medium or high confidence.

To quantify certainty in iNaturalist observations, a confidence score was developed, resulting in three levels of confidence: low, medium or high. Confidence is generated in three steps, accounting for uncertainty associated with the species, the media and georeferencing (Figure 1). The first step scores an observation considering the species being verified, with scores assigned based on whether the species has one or more easy‐to‐see diagnostic features and whether any other very similar species occur in the region. Secondly, scoring considers the media associated with the record, with higher confidence associated with clear images that show all the diagnostic features necessary for species identification. Finally, confidence is scored in relation to georeferencing, with positional accuracy of less than 1 km associated with higher confidence. The scores from each step are summed to produce an overall score of 4–8 (low confidence); 9–13 (medium confidence) or 14–18 (high confidence) (see step 4 of Figure 1). The total score for each of the three steps varies, reflecting that confidence associated with the species and media is affected by more factors than that associated with georeferencing. This variance is reflected by the number of levels in each step of the scoring process. If a ‘stop’ point is reached, confidence in the observation cannot be scored. This happens when: species are not morphologically distinct and molecular analyses are required to confirm identification, uploaded images are not clear enough to facilitate identification, images do not show the required diagnostic features or when geographical coordinates are not provided for the observation.

### 2.1 Marine Alien and Cryptogenic Species—South African Case Study

Alien species are recognised as major threats to natural ecosystems, causing the homogenisation of biota and contributing to approximately 60% of known recent extinctions (Roy et al. 2024). The implementation of long‐term standardised monitoring for alien species is essential for successful alien species management (Pyšek et al. 2020; Loureiro, Peters, and Robinson 2021). However, the intense monitoring required to support such evidence‐based management rarely occurs due to resource limitations (e.g., limited time, personnel and funding) (Delaney et al. 2008; Beric and MacIsaac 2015). This situation is often exacerbated in developing regions (Shackleton, Shackleton, and Kull 2019) where resources required to support monitoring are frequently limited (Evans and Blackburn 2020). Despite these limitations to monitoring, the need for accurate data on biological invasions persists, with the need for monitoring of marine alien species having been identified in South Africa (Loureiro, Peters, and Robinson 2021). Therefore, exploring cost‐effective methods of monitoring, such as utilising crowdsourcing through citizen science initiatives has the potential to bridge the gap between data requirements and scientific understanding (Encarnação, Teodósio, and Morais 2021; Garcia‐Soto et al. 2021; Howard et al. 2022). Citizen science observations have a great potential to complement research in terms of large geographical coverage and high species diversity (Hochmair et al. 2020). Therefore, in recognition of the threat that these species pose and the limited resources to monitor alien species in understudied regions, vital information such as the data provided in this case study has the potential to contribute to much needed on‐going research efforts.

Historical iNaturalist data (April 2008 to April 2023) were extracted utilising the explore page on iNaturalist (https://www.inaturalist.org/observations; accessed 19 April 2023). Searches were made for species previously recognised as alien (i.e., species whose presence in a region is attributable to human actions that enable them to overcome fundamental biogeographical barriers sensu Richardson, Pyšek, and Carlton 2011) and cryptogenic (i.e., species of unknown origin sensu Robinson et al. 2016) in South Africa. This included a total list of 137 species that span a wide range of taxa. Data extracted included the species name, image, positional accuracy and observation quality grade. The taxonomic and georeferencing accuracy of each observation was assessed and observations classified as correct, incorrect or uncertain. The identification of each observation was aided using primary literature sources that provided clear guidance on the morphometric identification of the species known as alien and cryptogenic in South Africa (e.g., taxonomic reference guides such as Darwin (1854), Monniot et al. (2001), Gibbons and Samaai (2005), Smith and Gordon (2011) and Rocha et al. (2019)). An identification was considered uncertain when the quality of images were too poor to determine identity to species level or if images did not show characteristics required to identify to species level. The uncertain category was also assigned for cryptic taxa that require molecular evidence for species‐level identification. The plausibility of georeferencing was considered for each observation; all records occurring on land were classified as incorrect and were excluded.

### 2.2 Statistical Analyses

All analyses were conducted in R version 4.3.1 (R Core Team 2023). The relationship between accuracy and confidence was assessed using a Kendall's rank correlation. The same analysis was used to access the association between iNaturalist quality grade and confidence, as well as between accuracy and quality grade. Following Schober, Boer, and Schwarte (2018) the strength of these relationships was assessed based on Tau.

## 3 Results

In total, 957 observations of 36 marine alien and cryptogenic species were extracted from iNaturalist. These species belonged to a variety of taxa, ranging from small crustaceans (e.g., amphipods and isopods) to green algae (Table 1). Mollusca had the highest proportion of observations and accounted for 45.4% of all extracted observations. 20.6% of observations were of species that were not scorable as they require molecular evidence for species‐level identification. All observations extracted from iNaturalist then underwent confidence scoring. In total, 226 observations (23.6%) received a high confidence score, 341 observations (35.6%) received a medium score, with 32 observations (3.4%) receiving a low confidence score. Importantly, 358 observations (37.4%) were not scorable due to poor media quality or the fact that the observations were of taxa that require molecular analysis to confirm species‐level identification. On average, confidence scoring took 1.5 min per observation.

**TABLE 1:** Alien and cryptogenic marine species for which observations were extracted from iNaturalist for South Africa.

Taxon	Taxon
**Porifera** (2.2)	**Mollusca** (45.4)
*Suberites ficus*	*Anteaeolidiella indica*
**Cnidaria** (0.4)	*Myosotella myosotis*
*Ectopleura crocea*	*Polycera hedgpethi*
*Pennaria disticha*	*Tarebia granifera*
*Obelia dichotoma*	*Thecacera pennigera*
**Annelida** (0.4)	*Martesia striata*
*Ficopomatus enigmaticus* ^*#*^	*Mytilus galloprovincialis*
**Arthropoda** (6.4)	*Semimytilus patagonicus*
*Anisolabis maritima*	**Chordata** (5)
*Amphibalanus venustus*	*Botryllus schlosseri*
*Balanus glandula*	*Ciona robusta*
*Megabalanus tintinnabulum*	*Clavelina lepadiformis*
*Ligia exotica*	*Cystodytes dellechiajei*
*Cymadusa filosa*	*Styela plicata* ^*$*^
*Melita zeylanica*	*Symplegma brakenhielmi*
*Carcinus maenas*	**Chlorophyta** (19.8)
**Bryozoa** (7.8)	*Codium fragile* ^*#*^
*Bugulina flabellata*	*Ulva lactuca* ^*#*^
*Virididentula dentata*	**Rhodophyta** (0.1)
*Watersipora subtorquata*	*Asparagopsis armata*
**Brachiopoda** (0.1)	**Tracheophyta** (12.4)
*Discinisca tenuis*	*Spartina maritima*
	*Stuckenia pectinata*

*Note:* Species for which internal morphology^$^ or genetic confirmation^#^ are required for a species‐level identification and could not be scored. The numbers next to headings indicate the percentage of observations in that taxonomic group.

Overall, 498 observations (52%) had been correctly identified, 126 identifications (13.2%) were incorrect and the taxonomic identification of 333 observations (34.8%) was uncertain. On average, verification of observations took 3.5 min.

**FIGURE 2:**
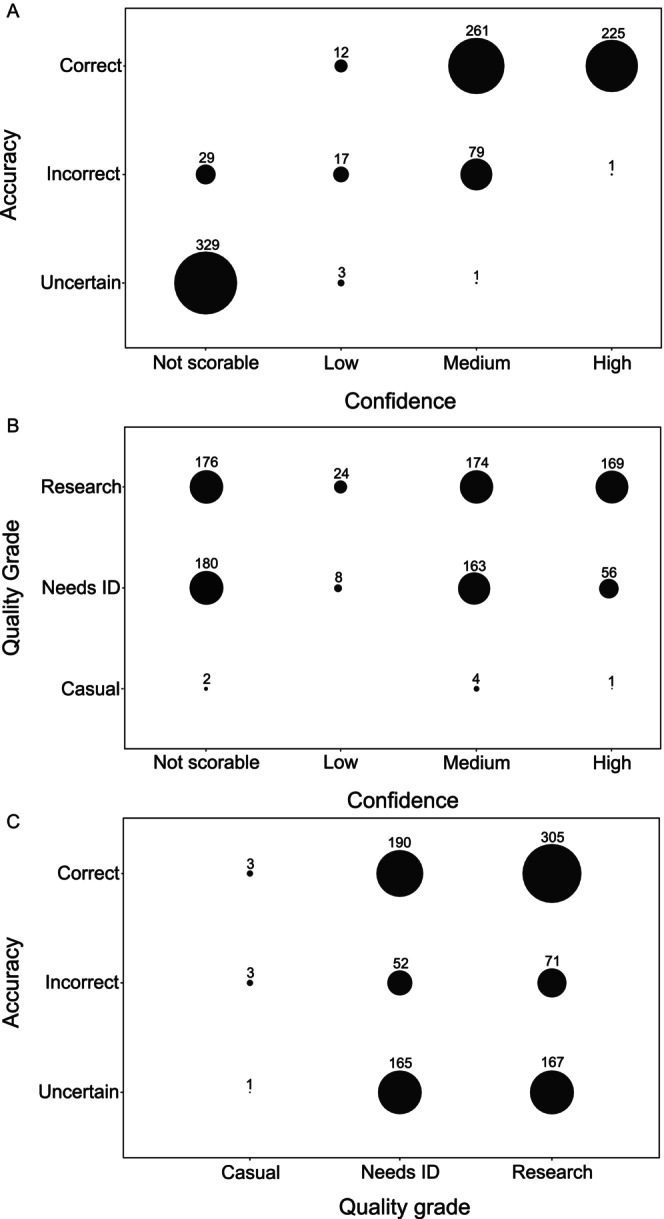
The proportion of observations within each confidence level in relation to (A) accuracy and (B) quality grade and (C) the proportion of observations within each accuracy and quality grade level. Note the size of the circles represents the percentage of observations within each category, while the number of observations are provided above each circle.

Accuracy and confidence were positively correlated (Kendall's rank correlation: *τ* = 0.82, *p* < 0.001; Figure 2A), and showed a strong relationship. Importantly, confidence could not be scored for almost a third of all observations. Of the 957 observations, 543 observations (56.8%) were graded as ‘research grade’ on iNaturalist. Additionally, 407 observations (42.5%) were assigned ‘needs ID’ with 7 observations (0.7%) graded as ‘casual’. iNaturalist quality grade and confidence were positively correlated (Kendall's rank correlation: *τ* = 0.16, *p* < 0.001; Figure 2B), as were quality grade and accuracy (Kendall's rank correlation: *τ* = 0.10, *p* < 0.05; Figure 2C). However, the strength of these relationships were weak and negligible, respectively. Although significant, the strength of the relationship between quality grade and accuracy is far weaker than that between accuracy and confidence, highlighting the value of the confidence protocol over the inherent iNaturalist quality grade. Interestingly, confidence could not be scored for a high proportion (18.4%) of observations that were classified as ‘research grade’ because observations were of cryptic taxa that require genetic confirmation of identification or image quality was too poor to make a sound identification.

## 4 Discussion

Unverified citizen science data has the potential to severely compromise the quality of resulting datasets (Falk et al. 2019). Data verification is therefore a crucial step in ensuring the flow of research‐quality data from citizen science sources. This study trialled a confidence score designed to quantify uncertainty within iNaturalist data. The confidence score was found to offer a cost‐effective and time‐efficient approach for identifying observations across a broad temporal and spatial scale. The application of this confidence score can thus help to identify high‐quality iNaturalist data for use in biological studies. Although the analysis for this paper was restricted to marine alien and cryptogenic species as a proof of concept, the confidence score should be applicable to all taxa.

Overall, observation confidence increased as accuracy increased, ultimately validating the confidence score. It is recommended that only observations scoring medium to high confidence should be retained for further analysis. This approach maximises the retention of accurate data. In this case study, 59.2% of extracted observations met this criteria. A large proportion of excluded observations were not scorable as these observations were of cryptic taxa that require molecular analysis to confirm species‐level identification. This highlights that despite the high proportion of data lost due to a lower confidence score, this loss is preferable to the inclusion of potentially inaccurate records.

Although some studies that utilise iNaturalist restrict data to observations that are graded as ‘research grade’ to ensure accuracy, this study found that this iNaturalist quality grade is an inadequate proxy for accuracy. This is reflected in 24.9% of observations listed as research grade being inaccurate or uncertain. While this study considered a variety of marine taxa, the generality of this finding across different systems remains untested. However, studies on lichens (Munzi, Isocrono, and Ravera 2023) and non‐marine molluscs (Barbato et al. 2021) have also highlighted inaccuracies in iNaturalist data, suggesting that the findings from this study could apply beyond marine systems. Restricting data usage to ‘research grade’ observations may exclude accurate observations if community engagement is low. This is because observations still requiring community agreement on the identification of the species are graded as ‘needs ID’. Other inaccuracies in quality grade are further inflated by individuals termed as ‘clickers’ (i.e., individuals wanting to score as many observations as possible regardless of their taxonomic knowledge (Barbato et al. 2021)) and by individuals who rely on the inbuilt machine learning process and accept identification suggestions without certitude (Munzi, Isocrono, and Ravera 2023). These caveats highlight that the use of ‘research grade’ data may introduce inaccuracy into datasets while excluding correctly identified observations, a situation that can be avoided through the use of the confidence score developed in this study. While the scoring process requires time to apply, the value of the protocol with respect to increased data accuracy validates its use instead of relying on the inherent iNaturalist quality grade.

Despite the strengths of the proposed scoring system, it is worth noting that it does require that those applying it hold foundational knowledge on the taxa being considered. This is because, although the specimen depicted in each iNaturalist record need not be identified by the researcher, users must hold taxonomic knowledge of the species to which the observation was allocated on iNtauralist in order to complete Step 1 (i.e., they need to know the diagnostic features of the species in question). As such, this confidence score compliments skilled researchers and will help them to ensure data quality data when utilising the valuable data held by iNaturalist.

One of the greatest advantages of citizen science initiatives is that data can be collected at the level of big data (Koo et al. 2022). However, if data are inaccurate and unreliable, the use of the data for research purposes remains limited. This problem is particularly worrisome as ‘research grade’ data is automatically uploaded to GBIF, with the contribution of these records increasing rapidly post 2011 (Contreras‐Díaz et al. 2023). As this database serves as a widely used global repository for species level data (Jetz et al. 2019), inaccuracies can negatively impact biodiversity conservation (Freitas et al. 2020; Contreras‐Díaz et al. 2023). As confidence scoring can easily be incorporated into research that aims to utilise data from citizen science initiatives, it is recommended that the confidence score proposed in this paper should become a standard requirement for ensuring data quality, rather than relying on the inherent iNaturalist quality grade. This recommendation is further supported by the data being better explained by the confidence score than the iNaturalist quality grade. Furthermore, the use of this confidence score reduces the time taken during the verification process and enables the retention of accurate data. This in turn could allow for the integration of readily available data into research and management streams while maximising the use of iNaturalist data for biodiversity studies.

## Author Contributions

**Sarah J. Ackland:** conceptualization (equal), formal analysis (lead), visualization (lead), writing – original draft (lead). **David M. Richardson:** conceptualization (equal), supervision (supporting), writing – review and editing (equal). **Tamara B. Robinson:** conceptualization (equal), project administration (lead), supervision (lead), writing – review and editing (equal).

## Conflicts of Interest

The authors declare no conflicts of interest.

### Open Research Badges

This article has earned an Open Data badge for making publicly available the digitally‐shareable data necessary to reproduce the reported results. The data is available at https://doi.org/10.5281/zenodo.13740672.

## Data Availability

The dataset, the description thereof and the associated code has been uploaded to Zenodo (https://doi.org/10.5281/zenodo.13740672).
